# Research on Chinese Consumers’ Attitudes Analysis of Big-Data Driven Price Discrimination Based on Machine Learning

**DOI:** 10.3389/fpsyg.2021.803212

**Published:** 2022-02-01

**Authors:** Jun Wang, Tao Shu, Wenjin Zhao, Jixian Zhou

**Affiliations:** School of Economic Information Engineering, Southwestern University of Finance and Economics, Chengdu, China

**Keywords:** big data, price discrimination, consumer attitude analysis, Labeled LDA, LSTM, Snow NLP

## Abstract

From the end of 2018 in China, the Big-data Driven Price Discrimination (BDPD) of online consumption raised public debate on social media. To study the consumers’ attitude about the BDPD, this study constructed a semantic recognition frame to deconstruct the Affection-Behavior-Cognition (ABC) consumer attitude theory using machine learning models inclusive of the Labeled Latent Dirichlet Allocation (LDA), Long Short-Term Memory (LSTM), and Snow Natural Language Processing (NLP), based on social media comments text dataset. Similar to the questionnaires published results, this article verified that 61% of consumers expressed negative sentiment toward BDPD in general. Differently, on a finer scale, this study further measured the negative sentiments that differ significantly among different topics. The measurement results show that the topics “Regular Customers Priced High” (69%) and “Usage Intention” (67%) occupy the top two places of negative sentiment among consumers, and the topic “Precision Marketing” (42%) is at the bottom. Moreover, semantic recognition results that 49% of consumers’ comments involve multiple topics, indicating that consumers have a pretty clear cognition of the complex status of the BDPD. Importantly, this study found some topics that had not been focused on in previous studies, such as more than 8% of consumers calling for government and legal departments to regulate BDPD behavior, which indicates that quite enough consumers are losing confidence in the self-discipline of the platform enterprises. Another interesting result is that consumers who pursue solutions to the BDPD belong to two mutually exclusive groups: government protection and self-protection. The significance of this study is that it reminds the e-commerce platforms to pay attention to the potential harm for consumers’ psychology while bringing additional profits through the BDPD. Otherwise, the negative consumer attitudes may cause damage to brand image, business reputation, and the sustainable development of the platforms themselves. It also provides the government supervision departments an advanced analysis method reference for more effective administration to protect social fairness.

## Introduction

Online consumption has become people’s everyday life, producing rich consumer data in significant dimensions, often referred to as big data in consumer markets. Big data promotes the vigorous development of the “Digital Economy,” convenient people’s lives, and satisfying consumers’ personalized needs for products and services. However, there are demonstrated and documented rejections of the intrusion of brands in private life, the abuse of data, and the fear that we are managed by algorithms (Lindstrom, 2016; Kotler et al., 2021). The consumers’ negative psychological perception of big data is not groundless. While big data drives companies to better meet consumers’ personalized needs, it also provides opportunities for price discrimination (PD). Existing studies have shown that e-commerce platforms usually require customers to confirm their identity through login. It provides a routine for companies to individually identify customers and link them to personal information such as purchase history, geographic location, age, and gender. Furthermore, e-commerce platforms can cross-refer this information with other shopping sites or social media data to more accurately estimate customers’ willingness to pay and determine the corresponding price. As a result, e-commerce platforms are likely to use their customer and third-party data to enforce PD for extra profits (Odlyzko, 2003). Typically, due to the application and popularity of big data technology, the differential pricing behavior based on consumers’ individual information is called Big-data Driven Price Discrimination (BDPD).

The BDPD not only abuses personal information but also violates the rights and interests of consumers (Li, 2020). As the product of the Internet economy, the BDPD has attracted worldwide attention from the government, media, academia, and even society. Scholars believe that PD, in the absence of supervision, will lead to potential unfairness and exploitation, especially in the online market (Papandropoulos, 2007). Furthermore, the BDPD is even more harmful to consumers with demand-inflating misperceptions and might reduce efficiency (Bar-Gill, 2020). A study by Favaretto et al. (2019) indicated that discriminatory consequences of data mining were mainly attributed to human bias and shortcomings of the law; therefore, the suggested solutions included comprehensive auditing strategies, implementation of data protection legislation, and transparency enhancing strategies. Both the United States and the European Union are committed to solving problems at the legal level. Moreover, many acts have been issued or are being formulated. However, due to the lack of in-depth research on the BDPD, many aspects still need to reach a consensus (Birget, 2017).

In recent years, it has also become one of the critical topics in China. However, most of the existing research focuses on legal (Fu, 2020; Liao, 2020; Wen and Mo, 2021) and qualitative analysis in economics (Lei et al., 2021; Wang, 2021; Xing et al., 2021). As for consumers’ attitudes quantitative studies, some institutes have provided classical questionnaire surveys. According to the survey results by The Beijing Consumers Association in March 2019, in a sample of 3,185, 88% of respondents believed that the BDPD is widespread, and 56% claimed to have experiences with it. The consumers also expressed a range of attitudes when they recognized the problem. According to the statistical result by I-Media Consulting 2018, in a sample of 1,164 samples, 77% of respondents cannot accept the BDPD behavior from e-commerce platforms, and 42% of netizens said they would consider substituting their apps. Furthermore, 39% of the surveyed respondents inform their family members about the BDPD behavior, and 25% compare it with other applications. Though the results of the two timely surveys are widespread, they are not satisfactory enough. The samples of surveyed respondents in each survey are small. The respondents were also limited to a narrow geographic and temporal range. The consumer’s attitude involves affect, cognition, and behavioral intention, and these three aspects can change with consumer’s experience. As a result, the above two studies mainly focused on limited surveys on whether the respondents were aware of, accepted BDPD, or replaced their APP application due to BDPD. Studies on the more granular aspects of consumers’ cognitive, behavioral intentions, affects, and relationships are lacking. However, in general, systematic research in this area is still lacking.

Yet, different from the traditional attitude questionnaire survey results, using social media comments to study has become a trend in recent years. Social media provides individuals with two-way communication and many-to-many information broadcasting. Social networks are becoming increasingly important to the public, businesses, and governments. The publics express their opinions and feelings through social media and their dissatisfaction and demands, which are deeply affected by social media. Companies are also relying more on social media to build customer relationships.

Moreover, with the help of Internet technology, social media channels are essentially accessible and scalable, where millions of consumers receive, disseminate, and generate information content (Brogan, 2010; Akar and Topcu, 2011). Researchers defined these as user-generated content or consumer-generated content in the context of social media (Pitt, 2012). The content generated by consumers through social media has guided scholars and practitioners to find possible ways to optimize marketing (Teng et al., 2015). Wang et al. (2019b) deconstructed the multifaceted dimensions of Chinese consumers’ image of boutique hotels with many online textual data from social media. Furthermore, Friedrich et al. (2019) and Wang et al. (2019a) excavated social media features as stimuli for consumers’ perceived utility value. A study analyzed the content of online Q&As texts to reveal the interactive impact of users’ attitudes toward products (Yan et al., 2020). By mining and analyzing the social medial comments data, it is possible to identify the consumers’ attitudes of the BDPD.

This article aims to combine machine learning models to construct a semantic recognition framework to study the affect-behavior intention-cognition consumers’ attitude toward the BDPD from social media comments. As Marquerie (2021) said, “The only thing that differentiates us from machines is fear.” This article is expected to discover what the publics fear in the cognition dimension, how strongly they fear in the affect dimension, and what they will do in the behavior dimension. Given this, it will help the government departments to further understand the public concern for the BDPD and their attitudes status. In this way, the targeted policies can be designed to supervise the BDPD event and protect consumers’ rights and interests. At the same time, based on the needs of the business’s sustainable development, platform enterprises can also fully evaluate and balance the relationship between additional profits brought by technology and brand image. Thus, the mutual trust relationship among enterprises, consumers, and the government can be established to eliminate the public’s fear of new technologies and machines and form a mutually beneficial situation.

The structure of this article is as follows:

Part 1: Introduce the related research of attitude theory and the machine learning model of this study.

Part 2: Design consumer attitude semantic recognition framework based on social media comments, build the machine learning model implementation process, and establish model results analysis method.

Part 3: Conduct empirical results and analysis to prove the feasibility of the method and obtain advanced suggestions and means for government management and enterprise operation.

## Backgrounds

### Consumer Attitude Theory

With the popularization and development of information technology, social media platforms such as Weibo, Zhihu, Toutiao, Xigua video, Tiktok, and Kuaishou have become new ways for consumers to obtain information, emotions and opinions expression, and clarify their attitudes. However, as a form of digital attitude expression, consumer-generated social media comments have no significant difference from traditional attitudes.

**FIGURE 1:**
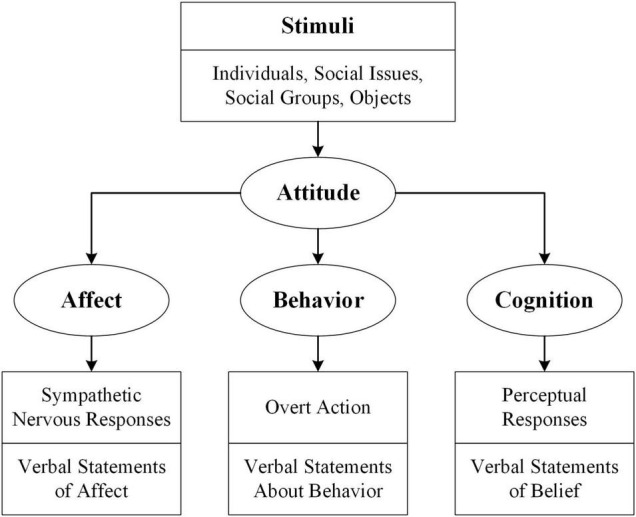
The tripartite model of attitude structure.

The study of consumer attitude refers to the research of consumers’ response to market information or marketing stimuli under specific trading situations. Some scholars believe that the consumer attitude is one-dimensional, which is people’s positive or negative emotional response to a particular object or relationship (Thurstone, 1927; Wightman, 1962; Shen, 1999). Other scholars believe that attitude consists of interweaving cognitive and affective factors (Kerch and Crutchfleld, 1948; Kim and Chan-Olmsted, 2005; Yi and Guo, 2009). However, some scholars believe that the consumer attitude is composed of three dimensions: affection, behavioral intention, and cognition, the famous ABC attitude theory (Rosenberg et al., 1960; Freedman et al., 1978; Sears et al., 1991) as shown in Figure 1. Some scholars have graphed the internal structural characteristics of attitudes dynamically. The main point is that attitude is a medium variable between stimulus and response, which are measurable independent and dependent variables, respectively. The three variables and their mutual relationship are conducive to the study of attitude measurement and attitude control (Breckler, 1984). Other scholars believe that the specific connotation of a cognitive factor in the “Three Elements” of attitude refers to people’s psychological impression of external objects, including relevant facts, knowledge, and belief, which is the basis of other parts of mentality. The specific connotation of emotional factors refers to people’s positive or negative evaluation of the object of attitude and the resulting emotions, such as respect and contempt, like and dislike, sympathy and indifference, etc. Thus, emotional factors are the core and key of attitude and affect cognition and behavioral tendency. The specific connotation of the behavioral intention factor refers to people’s reaction to the object of the consumer attitude. Still, the intention is not the behavior itself but the thought tendency before the specific behavior (You, 2018).

According to the definition of consumer attitude above, we believe that the online data of social media reflect consumers’ attitudes toward the BDPD, including three dimensions: affection, behavioral intention, and cognition. The consumers’ social media comments are often unstructured text. While text analysis tools are becoming more precise, they still need to be trained to understand key semantics and identify new ones. Therefore, qualitative research in related fields can guide machine learning. Simultaneously, facing the challenge of massive data, the introduction of machine learning methods is also necessary, which provide more accurate and timely quantitative analysis besides improving research efficiency.

### Semantic Recognition Based on Machine Learning

Currently, social media comments on the BDPD are primarily in the form of text. Therefore, automatic text classification has attracted wide attention as a critical technology for processing and organizing massive text data. A multi-label learning method is developed to identify better text data of social media comments, intuitively reflecting multiple semantic details of polysemy objects (Tsoumakas and Katakis, 2007; Tsoumakas and Zhang, 2009).

For the research target, the consumers’ concerns about the behavior and cognition corresponding to the BDPD must first be clustered from the original social media comment text data. In text-topic-mining, the Labeled Latent Dirichlet Allocation (LDA) model is widely used as clustering, and word analysis tool, such as scholar Li et al. (2008) have studied text clustering based on the Labeled-LDA model. In addition, Li et al. (2021) conducted a big-data analysis on the geographical relationship of the Arctic based on news reports. Unlike LDA, the Labeled LDA incorporates supervision by constraining the topic model to use only topics corresponding to a document label set (Ramage et al., 2009). Furthermore, the model constrains the topic model to use only those corresponding to the set of text labels that humans can observe for merger-supervised learning (Han et al., 2021). Given this, the Labeled LDA model is suitable for the study of this article.

The second process in this research is to classify the original comments to those obtained target topics about cognition or behavior intention. It is also the basis of the analysis of the relationships between topics. A few deep learning-based text classification models have been proposed to improve semantic information recognition and improve the classification efficiency of text, especially in Chinese text classification. Chen et al. (2015) constructed a method for Chinese text classification based on apparent semantics and latent aspects. Also, scholars tried to construct a novel Chinese short text classification method (Li et al., 2018). Zhao (2018) studied on short text feature expansion and classification based on topic model and deep learning. An efficient character-level and word-level feature fusion method for Chinese text classification was introduced by Jin et al. (2019). In some researches, hybrid neural networks were also used to Chinese short text classification (Wang, 2019). Further, many studies proved that the Long Short-Term Memory (LSTM) model and its improved models such as Bi-LSTM are performed well and efficiently in unstructured text multi-label classification, especially in Chinese unstructured text classification (Chen, 2021). Depending on the above previous studies, the LSTM model (Hochreiter and Schmidhuber, 1997) is applied to social media comment semantic recognition to implement the multi-label classification task in our research.

The sentiment analysis is an important part of this article according to the ABC theory of consumers’ attitudes. More importantly, it is helpful for the government and society to understand the public’s attitudes toward specific events, perceive public attitudes conversions, and make corresponding decisions. Sentiment analysis is the process of identifying users’ subjective emotions, opinions, and attitudes from text data (Mikolov et al., 2013).

The text sentiment analysis, also known as opinion mining, is defined as analysis, process, induction, and reasoning for subjectivity with emotion. Sentiment classification is a particular method of text classification.

Recently, the research of sentiment analysis mainly focused on two types of lexical-based and corpus-based methods. The lexical-based approach works by analyzing the composition of words and phrases that express positive or negative emotions. Scholars propose that this analytical method can preliminarily determine the emotional orientation of texts (Fu et al., 2017). However, it is unsuitable for all situations because emotion lexicons may have opposite emotional polarities in different contexts, insufficient to carry out objective emotion classification. The other corpus-based method, also known as the based machine learning method, can better complete text sentiment classification. The scholars have successfully applied many machine learning methods to predict the emotional orientation after the text is evaluated as words and converted into several features. The methods include Naive Bayes (Mukherjee and Bala, 2017), Support Vector Machine (Ye et al., 2009), Maximum Entropy (Catal and Nangir, 2017), and other machine learning techniques to predict the emotional orientation after the text is evaluated as words and converted into several features. For example, some scholars used supervised machine learning to classify WeChat text data (Zhang et al., 2018).

Bayesian classifiers Snow Natural Language Processing (NLP) of the Scikit-learn python machine learning library to analyze the sentiment of Chinese text are widely used. Scholar Chen and Liu (2020) found that the accuracy rate of sentiment classification was 89% by the Snow NLP Model, which exceeded the application of most classification algorithms in student evaluation of teaching texts. Researcher Zhang et al. (2020) also got satisfied sentiment results by the Snow NLP model to analysis e-commerce product comment data text. Abundant existing research results and support for personalized training make the Snow NLP model a robust choice for sentiment analysis in this article.

### The Research Works for Consumer Attitudes Toward the Big-Data Driven Price Discrimination Based on Social Media Comments

**FIGURE 2:**
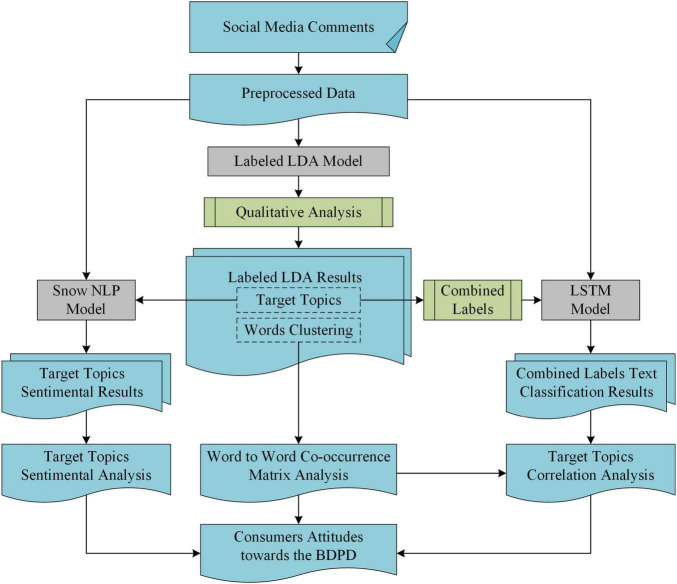
The research framework chart of consumer attitudes toward the Big-data Driven Price Discrimination (BDPD).

The research framework of the article is shown in Figure 2, which consists of three parts.

The first part is the collection and preprocessing of data sources. The relevant data were first obtained from social media platforms by the web spiders. Then, by preprocessing, the data were formed to the dataset for consumers’ comments toward the BDPD for this study.

Usually, consumers have different semantic expressions, and the attention of different perspectives toward the BDPD and the number of comments are quite numerous on social media. Therefore, it is necessary to build an automated semantic recognition framework for large-scale consumer comments. This research focuses on identifying what consumers know and care about, coping strategies, and consumer emotional orientation on the BDPD in the second part of this study with three steps. Under the guidance of previous studies, the first step is to cluster consumers’ concerned topics from their natural language texts. The machine learning model based on the Labeled LDA will be beneficial to complete this task. As an output of this task, the target topics and feature-word lists mined by Labeled LDA are also the basis of our follow-up works. Then, the target topics will be mathematically combined as the multi-label classification model labels based on LSTM. The second step is to classify consumer comments, and it can determine the target topics or combinations fit for consumer comments and their distribution by analyzing the classification results. Furthermore, analyzing the word-to-word co-occurrence matrix of feature word lists from Labeled LDA can splice the semantics from word pair level to sentence level. It can also confirm the rationality of target topics selection and explain the LSTM classification results to a certain extent. In the ABC theory of consumer attitude, emotional factors are also an essential part of consumer attitude. Therefore, in the third step of this part, the Snow NLP model is used for sentiment analysis of comments that have been classified into the target topics from Labeled LDA.

Combining qualitative and quantitative analysis, the target topics of this article includes two domains: consumer cognition and behavioral intention. As shown in Figure 2, the target topics are the key factors in this research. The researcher can achieve the distribution of consumers’ sentiment orientation in a finer granularity based on sentiment analysis results referring to the target topics. Similarly, the target topics and their combination guide the classification task to a finer granularity in multi-label classification. Based on the classification results, it is necessary to analyze the relationship between cognition and behavior, different cognitive aspects, and different behaviors. Thus, in the third part, this study hopes to verify the previous research results and common sense judgments quantitatively and hopes to find some relationships that have not been paid attention to or studied.

In summary, the semantic recognition framework in this article can identify consumers’ concerned topics about the BDPD and their emotional orientation and obtain the relationship between various topics. Thus, this study can get a quantitative, fine-granular, and multidimensional analysis of consumers’ attitudes toward the BDPD.

### The Machine Learning-Based Models for Semantic Recognition

The research works of this article are relied on three machine learning models as follows.

**FIGURE 3:**
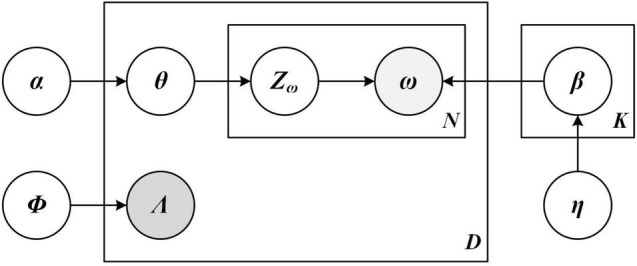
The Labeled Latent Dirichlet Allocation (LDA) model of semantic recognition framework.

The first one is the Labeled LDA Model. This study uses Labeled LDA model to cluster consumer comments and mine the topics of consumer comment texts. The specific algorithm principle of the labeled LDA model studied in this article is shown in Figure 3.

Assuming that each comment is composed of a series of words *w* and a binary topic indicator vector, a binary topic indicator vector of comment *d* is expressed as Λ^(*d*)^ = (*l*_1_, *l*_2_, ⋯, *l*_*k*_), where *l*_*k*_ ∈ {0,  1}, each component represents the existence and non-existence of the subject; The words in comment *d* are expressed as *w*^(*d*)^ = (*w*_1_, ⋯, *w_N_d__*), *w*_*i*_ ∈ { 1, ⋯, *V* }, where *N*_*d*_ represents the length of the comment text, *V* is the number of words, and *K* represents the total number of different tags in the corpus. The referential relationship of variables is shown in Table 1.

**TABLE 1:** The variables of the Labeled LDA model.

Symbol	Explanation
**β** *_*k*_*	The multinomial distribution parameter vector of the KTH topic
α	Dirichlet topic prior probability distribution parameters
η	Prior probability distribution of words
Φ_*k*_	The prior probability distribution of the label of the KTH topic
**Λ**	Binary (present/absent) topic indicator vector
***L*** ^(^ *^d^* ^)^	Projection matrix

**FIGURE 4:**
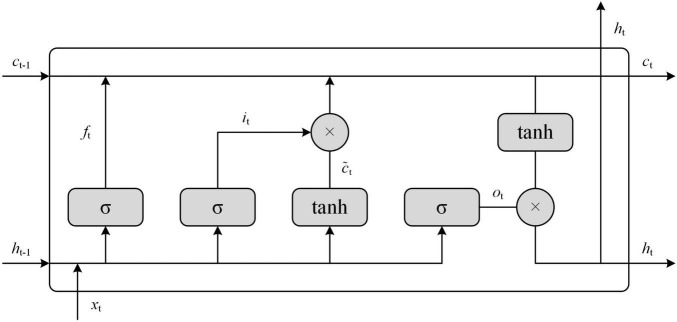
The Long Short-Term Memory (LSTM) model of semantic recognition framework.

The second model, LSTM, was chosen for the multi-label text classification task in this article. The core of LSTM is the cell state, which is updated by three gates of neurons: output gate, forget gate, and input gate. Based on this, the LSTM can control the transmission state through the gated form to achieve selective memory of information, retain important information, and forget unimportant details, as shown in Figure 4.

The specific algorithm of the LSTM model, in this article, is that each unit of LSTM has a forgetting gate to control the forgetting of *c*_*t–1*_ information. The sigmoid activation function is σ. The output of the forgetting gate has the same shape as the matrix of the *c*_*t–1*_ state. This matrix is multiplied point by point with *c*_*t–1*_ to determine the forgotten content. The forgetting gate output close to 0 is forgotten content, close to 1 is to be retained. The input of the forgetting gate *x_t_* is the input of the current moment, and *h*_*t–1*_ is the state of the hidden layer at the last moment. The calculation of forgetting gate is shown in Formula (1):


(1) $$f_{1}=\sigma \left(w_{f}\left[h_{t-1},x_{t}\right]+b_{f}\right)$$

The LSTM has forgettable gates and requires an input gate to store new information and create a new vector of candidate values $\tilde{c_{t}}$ to determine the updated values in the tanh layer. The input gates are also called *x_t_* and *h*_*t–1*_. The output of the updated information is a point-by-point product of $\tilde{c_{t}}$ I and *i_t_*, calculated by sigmoid function and are between 0 and 1, according to the formulas (2) and (3):


(2) $$i_{t}=\sigma \left(w_{i}\cdot \left[h_{t-1},x_{t}\right]+b_{i}\right)$$

(3) $$\tilde{c_{t}}=\text{tanh}\left(w_{c}\cdot \left[h_{t-1},x_{t}\right]+b_{c}\right)$$

Therefore, new memory cells come from the process of forgetting and remembering, as shown in formula (4):


(4) $$c_{t}=f_{t}*c_{t-1}+i_{t}*\tilde{c_{t}}$$

Finally, an output gate is needed to output another hidden state *h_t_* value as shown in formula (5) and (6):


(5) $$o_{t}=\sigma \left(w_{0}\left[h_{t-1},x_{t}\right]+b_{0}\right)$$

(6) $$h_{t}=o_{t}*tanh\left(c_{t}\right)$$

As this study’s third machine learning model, the Snow NLP was applied to analyze the emotion of comments under each target topic. The naive Bayes hypothesis is introduced in this study to avoid excessive feature combinations, which may lead to sparse data sets. Naive Bayes’ formula is:


(7) $$\text{P}\left(c_{j}/d\right)=\frac{P\left(c_{j}\right)P\left(d/c_{j}\right)}{P\left(d\right)}=\frac{P\left(c_{j}\right){∐}_{i=1}^{m}P\left(f_{i}/c_{j}\right)}{P\left(d\right)}$$

For our study, the denominator of (7) is fixed, because we only care about the relative size, so we only count the numerator. The estimates that will be obtained by model training are *P*(*c*_*j*_) and *P*(*f*_*i*_/*c*_*j*_):


(8) $$\hat{P}\left(c_{j}\right)=N_{j}/N$$

(9) $$\hat{P}\left(f_{i}/c_{j}\right)=\frac{\sum_{k=1}^{N}I\left\{c=c_{j}andf=f_{j}\right\}}{\sum_{k=1}^{N}I\left\{c=c_{j}\right\}}$$

The most fundamental feature of naive Bayes classifier is the introduction of the naive Bayes hypothesis, that is, the words in the document are conditional independent, which is a very strong assumption. It is not very common, but it works. In addition, naive Bayes has a “zero probability” problem in practical applications. To solve this problem, Laplace Smoothing technology is introduced. That is, when calculating the probability, some numbers will be added to the numerator and denominator so that it is not zero. When Laplace Smoothing is available, the formula is as follows:


(10) $$\hat{P}\left(f_{i}/c_{j}\right)=\frac{\sum_{k=1}^{N}I\left\{c=c_{j}andf=f_{j}\right\}+1}{\sum_{k=1}^{N}I\left\{c=c_{j}\right\}+H}$$

### Experimental Research and Analysis

#### Experimental Contents

The experiments in this study have three parts. The first part is target topics mining and feature words clustering for consumers’ attitudes toward the BDPD. By the inner method of the Labeled LDA model, it provides the feature word list. After that, the word-to-word co-occurrence matrix of the feature words combines qualitative analysis results to excavate the target topics of “Cognition” and “Behavioral Intention” domains in consumer attitudes. As a result, 8 related target topics were thus identified. In the second part, the 8 target topics are grouped into 256 labels for classification task. Then, by using the LSTM multi-label classification model, each comment data will be uniquely classified into a specific label, which solves the problem of classifying a comment involving multi-topics. Based on the above model, a sentence-level semantic quantitative analysis of consumer attitudes toward the BDPD was carried out. The third part is the sentiment analysis of each target topic based on the Snow NLP classification model. The output of the Snow NLP model is a score of 0–1. That is, the model calculates the probability that a comment data is a positive sentiment. In this research, the 0–1 is divided into three segments, indicating whether each comment is negative, neutral, or positive. By counting the sentimental score of comments under each topic, it can get the proportion of negative, neutral, and positive comments. Then the weighted average formula is used to calculate the sentimental intensity according to the overall proportion of all topics.

#### Data Collection

This study uses Python programming technology to capture 68,769 consumer online text comments about the BDPD on social media from January 1, 2018 to June 28, 2021, with third-party data sources including Weibo, Zhihu, Toutiao, Xigua video, Tiktok, and Kuaishou. This article chose these social media comments as data sources for the following reasons. Weibo is the leader of the social platform for sharing a brief, real-time information, founded in 2009, and has more than 340 million active users. Zhihu is the leader of a high-quality Q&A community on the Chinese Internet, has more than 2.5 million monthly active paying users, more than 3 million total content, and more than 3 billion annual visitors since 2011. Toutiao is the top 1 recommendation engine based on data mining and has 260 million active users monthly. Tiktok and Kuaishou are the top 2 short video publishing platforms in China, with 620 and 380 million daily active users, respectively. But more importantly, unlike the comment data on e-commerce platforms, these third-party consumer comments based on social media are more objective in commenting on individuals, events, social issues, social groups, and organizations. Obviously, social media reviews expressed the consumers’ perceptions of events, emotional feelings, which are unpleasant, hate, resentment, and consumers’ behavioral intentions such as whether to buy, protect themselves, and seek government regulation wills.

### Experimental Parameters Setup

After optimization processes in the experiments, the parameters of the three models applied in this study are finally set as follows:

The Labeled LDA model experimental setup: On the basis of data preprocessing, this article uses a python third-party module Scikit-learn commonly used in machine learning to train Labeled LDA subject model, and sets the super parameter alpha value to 0.5 and beta value to 0.1.

The LSTM model experimental setup: In this model, the word embedding vector dimension is 256, label vector dimension is 128, hidden layer dimension is 200, optimizer is Adam, batch size is 32, and dropout is 0.4.

The Snow NLP model experimental setup: On the basis of data preprocessing, this article uses a python third-party module Scikit-learn commonly used in machine learning to train Snow NLP model, and sets the super parameter alpha value to 0.5 and beta value to 0.1.

### Experimental Result and Analysis

#### The Labeled Latent Dirichlet Allocation Model Experiment Result and Analysis

The first part of the experiment result comes from the Labeled LDA model and consists of three components: qualitative analysis, target topics mining, and feature words clustering.

**FIGURE 5:**
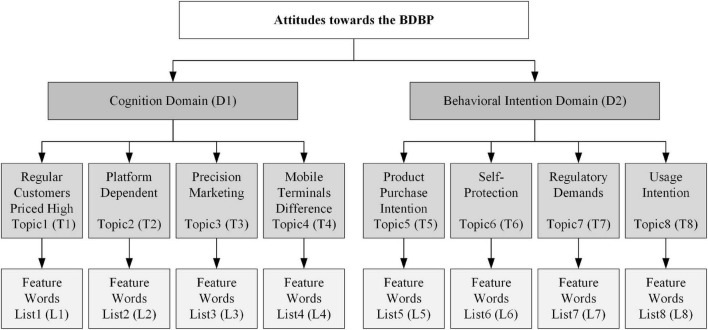
The composition chart of the Labeled LDA model experimental results.

**FIGURE 6:**
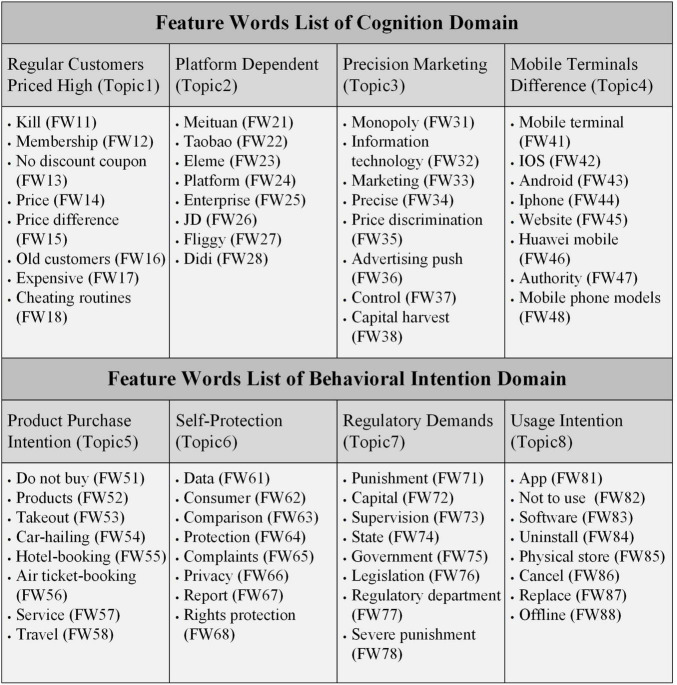
The feature word lists of the target topics.

The composition chart of the experimental results of the Labeled LDA model is shown in Figure 5. First, with the guidance of sociology, psychology, and management theories, this article conducts qualitative analysis on the preprocessed data. It divides consumer comments toward the BDPD into two major domains: “Cognition” and “Behavioral Intention.” Second, the previous qualitative analysis combined with the topics mining and feature words clustering results of the Labeled LDA model selected eight target topics belonging to these two domains and most expressed by consumers. In the “Cognition” domain, the four most representative target topics are “Regular Customers Priced High,” “Platform Dependent,” “Precision Marketing,” and “Mobile Terminals Difference.” In the “Behavior Intention” domain, the top 4 target topics are “Product Purchase Intention,” “Self-Protection,” “Regulatory Demands,” and “Usage Intention,” after analysis of the model results. Finally, through analyzing feature words clustering results of the Labeled LDA model, the feature word lists were created, which included the top 8 feature words under each target topic. It should be noted that for most Chinese consumers, the BDPD is an abstract academic concept, which will be expressed in a Chinese proverb, which literal translates to “Big-data Killing” or “Killing by Big-data.” Hence, in this article, the concept of the BDPD recognized by Chinese consumers is abstracted through the mining of four cognitive topics. Meanwhile, in Figure 6, the meaning of the feature words list about the eight target topics is the same as that of the glossary terms about the BDPD, presenting consumers’ social media comments on the BDPD.

As shown in Figure 5, the determination of target topics depends on qualitative analysis and feature words clustering results. Therefore, to more accurately explore the target topics about the BDPD in consumer attitudes, this study put the top eight feature words into the feature word list of each target topic, as shown in Figure 6.

In addition, through the word-to-word co-occurrence matrix, the relationship between the feature words of each target topic is analyzed and explained, which further proves that the target topic selection has not only the basis of qualitative but also the basis of quantitative analysis. However, each target topic has a corresponding word-to-word co-occurrence matrix data table in this article. Therefore, we do not show them one by one, but only selected two tables of “Regular Customers Priced High” (topic1) in the “Cognitive” domain and “Product Purchase Intention” (topic5) in the “Behavioral Intention” domain for display, as shown in Tables 2, 3.

**TABLE 2:** The word-to-word co-occurrence matrix date of “Regular Customers Priced High” (topic1).

Feature word	FW11	FW12	FW13	FW14	FW15	FW16	FW17	FW18
FW11	6585	395	702	489	835	1009	864	295
FW12	395	5433	1299	497	841	443	980	312
FW13	702	1299	5220	344	429	290	1316	326
FW14	489	497	344	5149	466	904	1052	301
FW15	835	841	429	466	4839	381	523	318
FW16	1009	443	290	904	381	3005	338	238
FW17	864	980	1316	1052	523	338	2905	275
FW18	295	312	326	301	318	238	275	2854
								

**TABLE 3:** The word-to-word co-occurrence matrix date of “Product Purchase Intention” (topic5).

Feature word	FW 51	FW 52	FW 53	FW 54	FW 55	FW 56	FW 58	FW 59
FW 51	7731	682	645	577	381	770	264	210
FW 52	682	2959	130	173	28	639	358	0
FW 53	645	130	2593	96	42	39	56	31
FW 54	577	173	96	1737	108	233	34	28
FW 55	381	28	42	108	1333	344	36	110
FW 56	770	639	39	233	344	1205	25	51
FW 57	264	358	56	34	36	25	622	14
FW 58	210	0	31	28	110	51	14	307

In Topic1, “Regular Customers Priced High,” by analyzing the data of the word-to-word co-occurrence matrix in Table 2, we observed that the feature word “Kill” appeared 6,585 times, ranking first, and co-appeared with “Old Customers” the most, 1,009 times. The second most frequently used word was “Membership,” and co-appeared with “No Discount Coupon” 1,299 times, ranking the first. Both the feature words “No Discount Coupon” and “Price” co-appeared with the feature word “More Expensive” the most, 1,316 and 1,052 times, respectively. The feature words “Price Difference” and “Membership” appear together 1,009 times at most, and “Cheating Routines” and “No Discount Coupon” appear together 326 times at most. By artificial reading, we will comment in the text “Old Customers” and “Membership” summarized into one word, “Regular Customers.” Then, combined with the feature words “Price Difference” and “No Discount Coupon,” using a sentence interpret is that customers have different prices because of the absence of discount coupons. In one word, there is a familiar pattern for PD to regular customers, who have no discount coupons or fewer from e-commerce platforms.

In Topic2, “Platform Dependent,” when consumers’ comments involve platform enterprises, the feature words “Meituan,” a takeout platform, appeared 5,174 times and co-appeared with “Eleme” 1,760 times. The shopping platform “Taobao” appeared 4,439 times and co-appeared with “JD” the most. “Fliggy,” an airline booking platform, and “Didi,” a taxi-hailing platform, also appeared 2,550 times and 2,240 times, respectively. Therefore, the platform-related topic of consumer cognition is closely related to people’s lives, including clothing, food, housing, and transportation.

In Topic3, “Precision Marketing,” the most consumer expression of this cognitive topic is “Monopoly” that appeared 1,326 times and co-appeared with “Precise” for 480 times. “Information Technology” and “Monopoly” were the second most frequently co-appeared, 338 times. The feature words “Marketing” and “Precise” co-occurred the most times. The feature words “Price Discrimination” and “Capital Harvest” co-appeared with “Monopoly” 270 times and 153 times, respectively. The co-occurrence of the feature word “Advertising Push” and “Precise” is the highest at 204 times. The feature words “Control” and “Information” co-appeared 173 times at most. It suggests that consumers’ cognition of the “Precision Marketing” topic is that information control, precision marketing or push, and PD are all monopoly harvesting methods.

In Topic4, “Mobile Terminals Difference,” according to collinear matrix data analysis, the feature word “Mobile Terminal” co-appeared with “IOS” 1,437 times, followed by “Android,” “Website,” and “Authority” for 301, 270, and 128 times, respectively. Finally, the feature words “Apple” and “Huawei” co-appeared 304 and 287 times with the model, respectively. Thus, based on the literature on big data, we interpret the topic “Mobile Terminals difference” as consumers’ cognition of the price difference of the same product and service between different mobile terminal systems such as “IOS” and “Android” and different models such as “Apple” and “Huawei.”

In Topic5, “Product Purchase Intention,” which is shown in Table 3, the consumers’ behavioral intention to buy products, the word “Do Not Buy” appeared most frequently 7,731 times, followed by the keyword “Products,” which appeared most frequently 2,959 times, and “Do Not Buy,” which appeared most frequently 682 times. Specific product descriptions are “Takeout,” “Car-hailing,” “Hotel-booking,” “Air Ticket-booking,” “Service,” and “Travel” in descending order of word frequency. Besides the co-occurrence of “Service” and “Product,” all these specific product names co-appeared with “Do Not Buy” for 645, 577, 381, 770, and 210 times, respectively, among which the highest co-occurrence frequency is not to buy air tickets. Thus, it suggests that the topic “Product Purchase Intention” expresses consumers’ behavioral intention of not buying products related to the BPDP.

In Topic6, “Self-Protection,” the feature word “Data” appeared 5,061 times and “Privacy” 1,390 times. The second feature word “Consumer” co-appeared with other five features, and “Data” co-appeared 355 times, followed by “Complaints” 292 times, “Protection” 261 times, “Rights protection” 184 times, and “Report” 162 times. It shows that the specific expression of consumers’ willingness intention to protect themselves is that data represent their privacy, and big data may expose their privacy. Consumers may protect themselves through complaints, reports, and rights protection action.

In Topic7, “Regulatory Demands,” the feature word “Punishment” appeared 2,800 times, ranking first, and “State” co-appeared the highest with 1,156 times. The word “Supervision” has the highest co-occurrence frequency with other words. And the word “Capital” has the highest co-occurrence frequency with 1,210 times, followed by “Regulatory Department” and “Government” with 815 times and 523 times, respectively. The words “Legislation” and “Severe punishment” also appeared 551 times in the comments. It shows that consumers’ desire for supervision is to call on the state and government departments to punish capital evil, the BDPD, and suggest that the best punishment method is legislation.

In Topic8, “Usage Intention,” the characteristic verb “Not to Use,” which expresses the intention of behavior, ranked the first with 1,056 times, followed by “Uninstall” 667 times, “Cancel” 378 times, and “Replace” 202 times. The feature word “APP” appeared the most frequently alone for 1,289 times and co-appeared with other six feature words. The word “Replace” appeared the most frequently for 824 times, followed by “Not to Use” 616 times. The co-occurrence matrix can indicate consumers’ behavioral intention to substitute or not use APPs involved in the BDPD. They may uninstall or cancel APPs, and they may choose physical stores or offline consumption.

#### The Long Short-Term Memory Model Experiment Result and Analysis

In the second part, the multi-label classification experiment results using the LSTM model. This experiment uses the four indicators to evaluate model performance. Among them, macro average composite index (MaF1) is the weighted average of macro accuracy (MaP) and macro average recall rate (MaR), all marked with (+), indicating that the higher the value of these three evaluation indexes, the better. The Hamming loss (HL) index is the number of classification labels that are wrong in predicting the evaluation model. In particular, (–) is used as the mark of this index, indicating that the lower the model’s prediction error value, the better.

Table 4 shows that the MaF1 value reaches 0.90, reflecting the excellent performance of the classification model from the precise. At the same time, the HL index value is 0.0012, indicating that the number of wrongly predicted tag pairs is small, and the classification model performance is better from the perspective of loss. It can also reflect the effectiveness of the multi-label classification model used in the quantitative semantic analysis.

**TABLE 4:** The performance of multi-label classification model.

Model	MaP(+)	MaR(+)	MaF1(+)	HL(–)
LSTM	0.92	0.89	0.90	0.0012

Then, to further analyze the results of the multi-label classification experiment, this study selected the top 30 classification results for discussion, as shown in Table 5. It can be seen from the table that about 25% of consumer comments do not involve any topics. In other words, the consumers only express their sentiment to the BDBP through text comments on social media, such as “I am furious when I meet the BDPD.”

**TABLE 5:** Experiment results of multi-label classification model.

	Code	Proportion (%)	Description
1	0	25.395	Only sentiment expression, not involving topics.
2	4	9.568	Topic2
3	1	8.294	Topic1
4	5	6.921	Topic1 + Topic2
5	69	3.949	Topic5 + Topic2 + Topic1
6	16	3.882	Topic7
7	68	2.981	Topic5 + Topic2
8	64	2.038	Topic5
9	101	2.026	Topic5 + Topic6 + Topic2 + Topic1
10	65	1.997	Topic5 + Topic1
11	35	1.724	Topic6 + Topic3 + Topic1
12	33	1.534	Topic6 + Topic1
13	20	1.385	Topic7 + Topic2
14	37	1.340	Topic6 + Topic2 + Topic1
15	34	1.149	Topic6 + Topic3
16	18	1.133	Topic7 + Topic3
17	97	1.083	Topic5 + Topic6 + Topic1
18	128	0.918	Topic8
19	132	0.872	Topic8 + Topic2
20	39	0.781	Topic6 + Topic2 + Topic3 + Topic1
21	21	0.773	Topic7 + Topic2 + Topic1
22	2	0.748	Topic3
23	109	0.736	Topic5 + Topic6 + Topic4 + Topic2 + Topic1
24	32	0.690	Topic6
25	17	0.624	Topic7 + Topic1
26	41	0.612	Topic6 + Topic4 + Topic1
27	133	0.595	Topic8 + Topic2 + Topic1
28	40	0.583	Topic6 + Topic4
29	197	0.546	Topic8 + Topic5 + Topic2 + Topic1
30	129	0.504	Topic8 + Topic1
–	–	–	Those accounting for <0.5% are omitted.

According to the results shown in Table 5, the “Platform Dependent” (topic2) accounts for 9.568%, ranking the first among the labels with a sole topic. Combined with the word-to-word co-occurrence matrix analysis, it can be seen that most consumers describe the BDPD, including takeout, shopping, ticket-booking, car-hailing platform, etc., through social media comments. Thus, it expresses that consumers’ cognition toward the BDPD highly correlates with the e-commerce platforms.

In addition, the top one pairwise combinatorial topic in consumer comments is the “Platform Dependent” and “Regular Customers Priced High” (topic2 + topic1), accounting for 6.921%. It can be explained that most of the consumers believe that various platform enterprises involve the BDPD. The primary way the e-commerce platforms enforce the BDPD is that regular customers do not have coupons, which leads to the price being more expensive than new customers, based on the word-to-word co-occurrence matrix analysis.

This experimental results also show that more than 49.35% of consumers can connect more than two topics, such as the permutation and combination of “Product Purchase Intention,” “Platform Dependent,” and “Regular Customers Priced High” (topic5 + topic2 + topic1), accounts for 3.95%, and the combined four topics “Product Purchase Intention,” “Self-Protection,” “Platform Dependent,” and “Regular Customers Priced High” (topic5 + topic6 + topic2 + topic1) accounts for 2.03%, etc. Based on the above word-to-word co-occurrence matrix analysis, the connections between multiple topics reveal that some consumers have an acute and profound perception of the BDPD. When most consumers recognize the platform enterprises involved in the BDPD, the consumer behavior intention is to refuse to buy the platform products. In addition, the consumers may raise self-protection awareness, which is the complaint, accusation, and rights protection.

It should be noted that in Table 5, the “Regulatory Demands” (topic7) account for 3.88%. The related combination topics, including the combined two topics, which is “Regulatory Demands” and “Platform Dependent” (topic7 + topic2), account for 1.36%, “Regulatory Demands” and “Precision Marketing” (topic7 + topic3) account for 1.13%, and “Regulatory Demands” and “Regular Customers Priced High” (topic7 + topic1) account for 0.62%. The combined three topics, which are “Regulatory Demands,” “Platform Dependent,” and “Regular Customers Priced High” (topic7 + topic2 + topic1), account for 0.77%. The statistics show that more than 8% of consumers have regulatory demands on market regulatory rules of the national and legal level. It shows that sufficient consumers lost confidence through negotiations with platforms to solve the BDPD dispute and urgently needed government regulation and legal protection.

Finally, this experimental results show that there are only 55 combined topics that contain “Self-Protection” and “Regulatory Demands” (topic6 + topic7). Thus, it can be explained that consumers rarely express the willingness to combine regulatory demands and self-protection. In other words, once consumers express their willingness to regulatory demands, they will rarely express their willingness to self-protect, forming two groups.

#### The Snow Natural Language Processing Model Experiment Result and Analysis

**FIGURE 7:**
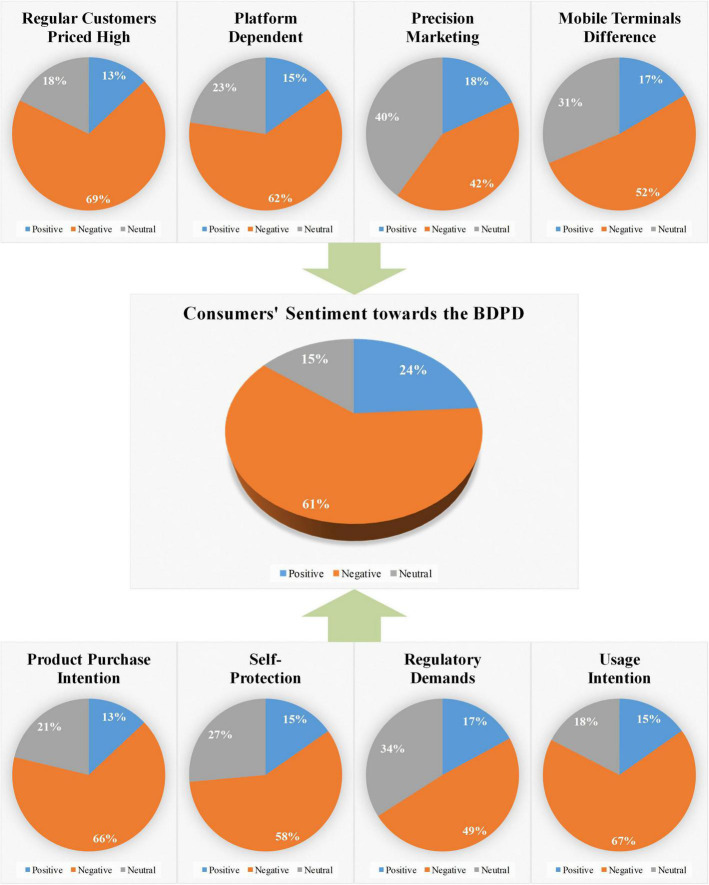
The experiment result of multi-topic sentiment classification.

The third part of the experiment results, the multi-topic sentiment classification experiment, identifies consumers’ sentiment toward the BDPD. The results are visualized as shown in Figure 7. It can be seen from the figure that the sentiment classification pie graph of consumers’ sentiment toward the BDPD is in the middle. The classification pie graph of the other eight topics is located above and below it, with arrows pointing to it, respectively. It intuitively shows that its classification results are calculated from the other eight classification results. The gray part represents neutral sentiment in the nine pie graphs, the blue part represents positive sentiment, and the orange part represents negative sentiment.

As shown in Figure 7, the pie graph of consumers’ sentiment toward the BDPD shows that the consumers’ positive sentiments account for 24.01%, negative sentiments account for 61.25%, and neutral sentiments account for 14.74%. Thus, the proportion of negative sentiment is far more significant than the neutral and positive sentiment, indicating that most consumers expressed negative sentiments toward the BDPD through social media comments.

Also, in Figure 7, the top three about the orange part, which represents negative sentiment in the pie graph of eight topics, is “Regular Customers Priced High” (topic1), “Usage Intention” (topic8), and “Product Purchase Intention” (topic5). Combining the semantic recognition experimental results, we found that most of the consumers express the negative sentiment toward “Regular Customers Priced High.” In other words, consumers’ objectionable impression about the BDPD is mainly reflected in topics of cognition aspects. Then, the consumers’ behavioral intentions include the topic “Usage Intention” and “Product Purchase Intention,” expressing highly negative sentiment. That is to say, platform enterprises involved in the BDPD may lead to consumers’ lower behavioral intentions with usage and purchase.

Finally, it can be seen from Figure 7 that the biggest gray and blue part in the eight pie graphs are both shown in the precision marketing pie graph. Thus, it shows that consumers have less negative sentiments about “Precision Marketing” (topic3). The reason may be that platform enterprises’ precision marketing technology helps consumers to select goods and services conveniently and effectively.

## Conclusion

In this study, we reviewed the classic consumer attitude theory, approached the theory from a new perspective, which is a method that includes a semantic recognition framework with multi-topic sentiment analysis by using machine learning models. Then, based on the consumers’ attitudes toward the BDPD, expressed by social media comments, this article has conducted comprehensive research on the consumers’ attitudes. It contains three dimensions: affection, behavior intention, and cognition toward the BDPD, and achieved good empirical results that reflect the practicability of the new perspective in the consumer attitude theory research.

Based on the above research results, we summarize some theoretical and practical contributions. First, unlike the traditional quantitative analysis method based on questionnaire data, we used social media data and conducted multidimensional and fine-granular quantitative analysis of consumers’ attitudes through machine learning models. As a result, the quantitative results are more objective, detailed, and accurate. Second, through our experiments, the collected Chinese text data about consumer attitudes and the trained machine learning models will provide a new research foundation for future researchers to study consumer attitudes. Finally, this study also has some practical enlightenment for relevant market operators and regulatory authorities. The results show that, in the face of the BDPD, the consumers choose to call on government departments to supervise and severely punish the enterprise platform involving the BDPD. Alternatively, they would try to use some means to self-protection such as complaints, reports, and rights protection action. Thus, the government regulation is still necessary when self-awareness and self-discipline cannot be guaranteed for all enterprises. The empirical results also show that consumers express their behavioral intention to BDPD-related platform companies in comments, such as not buying products, uninstalling or deleting APPs, and choosing physical stores or offline consumption. This kind of behavior or intention leads to damages for the corporate image, integrity, and reputation, impeding the sustainable development of the platform itself, further leading to chaos in the consumer market, and affecting the digital economy’s development. In summary, this study’s practical contribution helps the government to obtain consumers’ attitudes and formulate corresponding regulatory measures quickly. In addition, our research results can provide a new reference for the government to regulate the consumer market. Meanwhile, the results remind the e-commerce platforms to pay attention to the potential harm to consumers’ psychology while bringing additional profits through the BDPD.

Although the existing research results have been contributed, there are still areas for improvement. First, in terms of data collection, this article collected only the text data about consumers’ attitudes toward the BDPD. Although the data source after preprocessing is highly credible, the data range is relatively small, needs to be enriched, and enhanced. Second, both topic classification and sentiment analysis models need to strengthen training on the premise of further enriching the data to improve the classification accuracy, then, based on model improvement, fine-granular research on consumer attitudes, including sentiment, behavior intention, and cognition. In the future, based on the advantages of the machine learning model, we will study the evolution trend of consumer attitudes by introducing the timeline.

## Data Availability Statement

The original contributions presented in the study are included in the article/Supplementary Material, and further inquiries can be directed to the corresponding author.

## Author Contributions

TS and JZ developed the theoretical framework and model in this study and drafted the manuscript. JW and WZ implemented the algorithm and performed the experiments and result analysis. All authors contributed to the article and approved the submitted version.

## Conflict of Interest

The authors declare that the research was conducted in the absence of any commercial or financial relationships that could be construed as a potential conflict of interest.

## Publisher’s Note

All claims expressed in this article are solely those of the authors and do not necessarily represent those of their affiliated organizations, or those of the publisher, the editors and the reviewers. Any product that may be evaluated in this article, or claim that may be made by its manufacturer, is not guaranteed or endorsed by the publisher.
